# Fabrication of Highly Sensitive Capacitive Pressure Sensors Using a Bubble-Popping PDMS

**DOI:** 10.3390/polym15153301

**Published:** 2023-08-04

**Authors:** Yunseok Jang, Jeongdai Jo, Seung-Hyun Lee, Inyoung Kim, Taik-Min Lee, Kyoohee Woo, Sin Kwon, Hyunchang Kim

**Affiliations:** Department of Printed Electronics, Korea Institute of Machinery & Materials, Daejeon 34103, Republic of Korea

**Keywords:** capacitive sensors, bubble, bubble-popping, PDMS, pressure sensor

## Abstract

Attempts have been made to introduce microstructures or wrinkles into the elastomer surface to increase the sensitivity of the elastomer. However, the disadvantage of this method is that when a force is applied to the pressure sensor, the contact area with the electrode is changed and the linear response characteristic of the pressure sensor is reduced. The biggest advantage of the capacitive pressure sensor using an elastomer is that it is a characteristic that changes linearly according to the change in pressure, so it is not suitable to introduce microstructures or wrinkles into the elastomer surface. A method of increasing the sensitivity of the capacitive pressure sensor while maintaining the linearity according to the pressure change is proposed. We proposed a bubble-popping PDMS by creating pores inside the elastomer. The sensitivity of the pressure sensor made of the bubble-popping PDMS was approximately 4.6 times better than that of the pressure sensor without pores, and the pressure sensor made of the bubble-popping PDMS showed a high linear response characteristic to the external pressure change. These results show that our pressure sensor can be used to detect applied pressures or contact forces of e-skins.

## 1. Introduction

Flexible sensors have many advantages over rigid sensors with a great potential for electronic skins (e-skins), wearable devices, and flexible sensors as it is reported as light weight, with a high flexibility, good resolution, and fast response [1,2]. Among the various flexible sensors, the flexible pressure sensor is receiving a lot of attention because it can measure a rather weak pressure signal, such as a human blood pulse, heart rate, and respiration [3]. Pressure sensors are important because the pressure inside the body, such as the brain, blood, and organs, can provide people with indirect and important information about major diseases at the lowest cost [1,4].

A pressure sensor is a kind of electronic device that can convert a pressure signal into a corresponding electrical signal. In pressure sensors, pressure changes are mainly converted into resistive, piezoelectric, triboelectric, and capacitive electric signals [5,6]. Pressure sensors based on resistive, piezoelectric, and triboelectric characteristics are both generally and highly nonlinear, whereas the capacitive pressure sensor has a good linear response characteristic [7,8]. The capacitance per unit area for a capacitive sensor is given in the following equation:

(1) $$C=\frac{\varepsilon_{r}\;\varepsilon_{o}}{d}$$

where $\varepsilon_{r}$ is the relative dielectric constant of the dielectric layer, $\varepsilon_{o}$ is the permittivity of free space, and ***d*** is the thickness of the dielectric layer [9]. This equation shows that the capacitance per unit area is in an inverse proportion to the thickness, and the capacitive pressure sensor has a good linear response to the external stimuli. 

Since the elastomer is a material with a large change in thickness according to the external stimuli, polydimethylsiloxane (PDMS) was used as the dielectric layer in this study. To make a sensitive sensor for a good signal-to-noise ratio, the thickness must change easily when exposed to weak forces. Some studies have conducted the creation of pores on the elastomer surface to increase the sensitivity to the external stimuli [10,11]. The pores on the surface provide a microscopic space where the elastomer can deform when pressed by an external force, increasing the sensitivity of the sensor. To create pores on the elastomer surface and improve the sensitivity [12,13], Z. Bao et al. used an array of pyramidal microstructured elastomers [14], and H. S. Lee et al. used a micro-wrinkled elastomer [15]. When external pressure was applied to these microstructures or wrinkles, the contact area of the electrode, in contact with the microstructures or wrinkles, increased due to the deformation of the elastomer. This increase in the contact area affected the capacitance and deteriorated the linear response characteristics.

In this study, we proposed a method to form pores inside the elastomer to increase the sensitivity while maintaining the linear response characteristics and to eliminate the effect of contact area changes that occur when force is applied. A bubble-popping PDMS has been proposed to form pores inside the elastomer. In order to confirm the pore effect inside the PDMS, the characteristics of the PDMS without pores were also compared.

## 2. Materials and Methods

### 2.1. PDMS with Pores inside the Elastomer

The sheets with pores inside the elastomer were prepared using Polydimethylsiloxane (PDMS, Sylgard 184 silicon elastomer kit, Dow Corp., Midland, MI, USA) [16] and a water emulsion. The PDMS and water emulsion was made with a planetary mixer (Thinky Corp., Laguna Hills, CA, USA). The weight ratios of PDMS/hardener/water were 10:1:0, 10:1:0.1, 10:1:0.5, and 10:1:1, respectively. When the water was more than the 10:1:1 ratio, a stable emulsion could not be formed, and excess water was floating on the PDMS and water emulsion. Based on this, the 10:1:1 ratio was determined to be the maximum ratio that could be emulsified with water. Various proportions of the PDMS and water emulsions were coated onto the polyimide sheets using an applicator coater with 700 μm spacing, and then cured on a hot plate at 100 °C for 30 min. Residual water in the PDMS sheet was removed using the storage in a vacuum oven at 60 °C for one day.

### 2.2. Characteristics of Pressure Sensors Made from Various PDMS Sheets

The pressure sensor was made by placing various PDMS sheets between a circular SUS electrode with dimensions of 1.14 mm × 1 mm (i.e., diameter × height, top electrode) and a square brass electrode with dimensions of 30 mm × 30 mm × 15 mm (i.e., length × width × height, bottom electrode). The rigid SUS electrode was used as the upper electrode to prevent the deformation of the electrode during measurement. An Agilent 4980A Precision LCR meter was used to measure the capacitive responses of the various PDMS pressure sensors to the various stimuli. The pressure sensing capabilities of the sensors were characterized using a computer-controlled homemade sensor measurement system with a z-axis moving stage (LNR50SE, Thorlabs Corp., Newton, NJ, USA) that had a movement range of 50 mm, a force gauge, and (M7-05, Mark-10 Corp., Copiague, NY, USA) a capacity of 250 gF. The moving stage moved at a speed of 1 mm/s. The load applied to the force gauge was measured, and the corresponding pressure was calculated by dividing the area of the top electrode.

## 3. Results and Discussion

**Figure 1:**
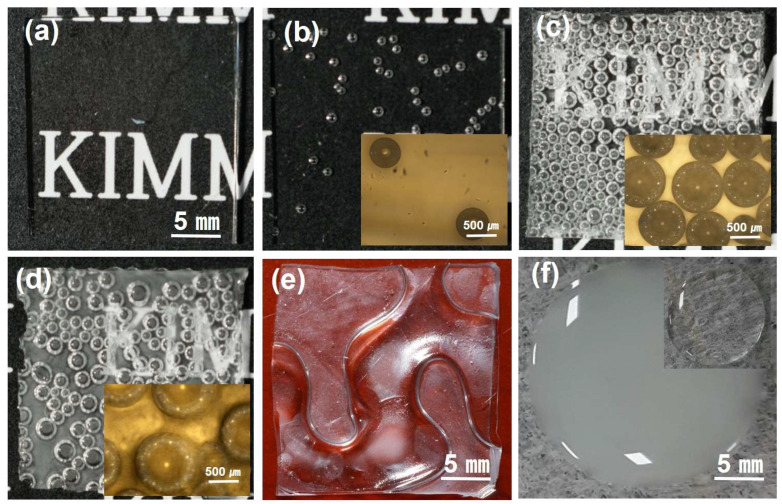
Digital camera images of the sheet of (**a**) 10:1:0, (**b**) 10:1:0.1, (**c**) 10:1:0.5, and (**d**) 10:1:1 ratios. Digital camera images of (**e**) the sheet made at 130 °C, and (**f**) the PDMS and water emulsion droplet. Inset: Optical microscopy images of the sheets with (**b**) 10:1:0.1, (**c**) 10:1:0.5, and (**d**) 10:1:1 ratios. Digital camera image of (**f**) the PDMS droplet in a 10:1:0 ratio.

Although the PDMS and water are immiscible, an emulsion containing fine water droplets in the PDMS can be prepared by mixing a small amount of water with a planetary mixer. This PDMS and water emulsion eventually phase-separated over time, but a stable emulsion was maintained while making the sheets with pores inside the PDMS. As shown in Figure 1f, the PDMS becomes white when emulsified with water. The pores were controlled by adjusting the amount of water, and the weight ratios of PDMS/hardener/water were 10:1:0, 10:1:0.1, 10:1:0.5, and 10:1:1, respectively. As shown in Figure 1, it can be seen that the size of the pores increased as the water content increased. The mean pore size of 10:1:0.1, 10:1:0.5, and 10:1:1 were 238 ± 22, 312 ± 97, and 467 ± 86 μm respectively.

Since the water vapor pressure of 3.2 kPa at 25 °C increased to 101 kPa at 100 °C [17], when the coated emulsion was placed on a hot plate of 100 °C, the water droplets in the emulsion evaporated quickly, creating bubbles where and some of them came out of the PDMS, but at the same time, the PDMS was cured and the viscosity rose quickly [18]. As the viscosity increased, some of the pores generated did not come out and were trapped inside the PDMS. This process is fast-paced and is similar to popping popcorn. Therefore, the PDMS made in this way is called a bubble-popping PDMS. If the temperature of the hot plate is as low as 70 °C, the bubbles escape before curing, so no bubble-popping PDMS was made. Conversely, if the temperature of the hot plate is as high as 130 °C, the pores came out faster with greater pressure and coalesced together to make them larger. The curing process of the PDMS would also be accelerated at same time. Finally, it formed a deep valley, as shown in Figure 1e. As a result, the pore size inside the PDMS is determined by the balance between the increased water vapor pressure inside the PDMS and the water vapor that escaped from the PDMS due to the increased water vapor pressure. Bubbles inside the PDMS can change the thickness of the PDMS because the bubbles have pore volume. The thickness of the bubble-popping PDMS was 687 ± 8, 927 ± 11, 915 ± 18, and 921 ± 25 μm at ratios of 10:1:0, 10:1:0.1, 10:1:0.5, and 10:1:1, respectively. The thickness of the bubble-popping PDMS at various ratios is similar, that is, the balance between the increased pore size, due to the vapor pressure increase, and the water vapor that escaped from the PDMS.

**Figure 2:**
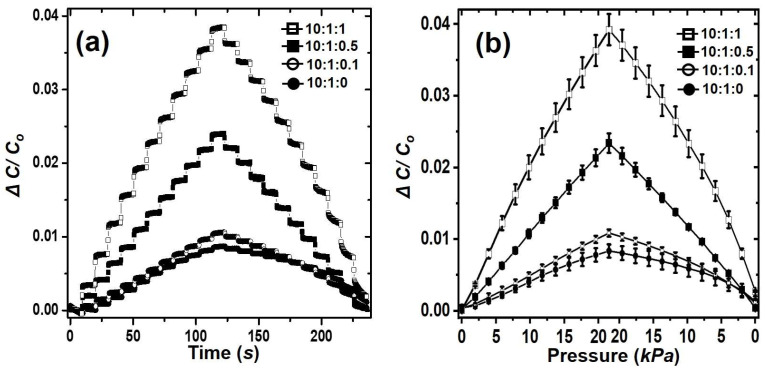
Relative capacitance change (Δ*C*/*C*_0_) as a function of (**a**) the step-by-step force in the 0~21.56 kPa range and (**b**) the normal force for the sheets with 10:1:0, 10:1:0.1, 10:1:0.5, and 10:1:1 ratios.

The step-by-step pressure sensor performance of the bubble-popping PDMS with various water contents was evaluated for forces from 0 to 21.56 kPa. As shown in Figure 2a, the relative capacitive change (Δ*C*/*C*_0_) was measured while applying a step force to the bubble-popping PDMS between the top electrode and the bottom electrode for 10 s, in which the relative capacitive change (Δ*C*/*C*_0_) increased as the step force increased. As the thickness of the bubble-popping PDMS decreased as the step force increased, the capacitance increased according to Equation (1).

The pressure sensitivity, defined as the *G-factor*, of the bubble-popping PDMS pressure sensors were determined by the slope of the relative capacitive change (Δ*C*/*C*_0_) versus the normal pressure [11,19]. As shown in Figure 2b, the pressure sensitivities increased with the amount of water, and the *G-factors* of 10:1:0, 10:1:0.1, 10:1:0.5, and 10:1:1 were 3.9 × 10^−4^, 5.1 × 10^−4^, 1.1 × 10^−3^, and 1.8 × 10^−3^ kPa^−1^, respectively. The bubble-popping PDMS with pores improved the sensitivity by approximately 4.6 times compared to the PDMS without pores. Thus, the response or sensitivity to the external pressure was significantly improved because the modulus of the bubble-popping PDMS decreased with the decreasing density of the bubble-popping PDMS due to pore formation inside the PDMS [20]. The density of 10:1:0, 10:1:0.1, 10:1:0.5, and 10:1:1 were 959 ± 3, 626 ± 4, 616 ± 2, and 522 ± 10 kg/m^3^, respectively. Although the sensitivity of our sensor is lower than that of the previously reported highest sensitivity (i.e., 0.55 kPa^−1^ from Z. Bao et al.) [21], previously reported sensors are nonlinear [21,22,23], while our sensors have a very high linearity except for the 10:1:1 ratio. In the case of the 10:1:1 ratio, many large pores formed inside the PDMS, which affected the elastic deformation of the PDMS and caused a slight slope change at about 11.76 kPa, as shown in Figure 2b. This linearity and improved sensitivity showed that the bubble-popping PDMS is useful as the dielectric layer for the capacitive pressure sensor.

**Figure 3:**
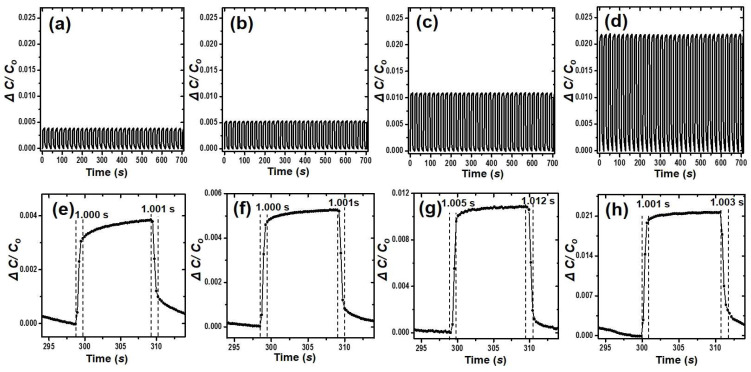
Durability test under an applied pressure of 11.76 kPa over more than 35 cycles for the sheets with (**a**) 10:1:0, (**b**) 10:1:0.1, (**c**) 10:1:0.5, and (**d**) 10:1:1 ratios. Pressure responses during single cycles, showing the response and release times for the sheets with (**e**) 10:1:0, (**f**) 10:1:0.1, (**g**) 10:1:0.5, and (**h**) 10:1:1 ratios.

The operating reliabilities, response times, and release times of pressure sensors made from the bubble-popping PDMS are shown in Figure 3. The device durability was obtained by measuring the relative capacitor change (Δ*C*/*C*_0_) of the devices over 33 repeated load/unload cycles with an applied pressure of 11.76 kPa. As the amount of water in the PDMS increased, the pressure sensitivity of the pressure sensors made from the bubble-popping PDMS increased. It showed the relative capacitor changes (Δ*C*/*C*_0_) of 0.0036, 0.0052, 0.011, and 0.022, respectively. The response and release times for the pressure sensors made from the bubble-popping PDMS are comparable, about 1 s. These results show that the pores of the bubble-popping PDMS improve sensitivity without altering the response and release time.

**Figure 4:**
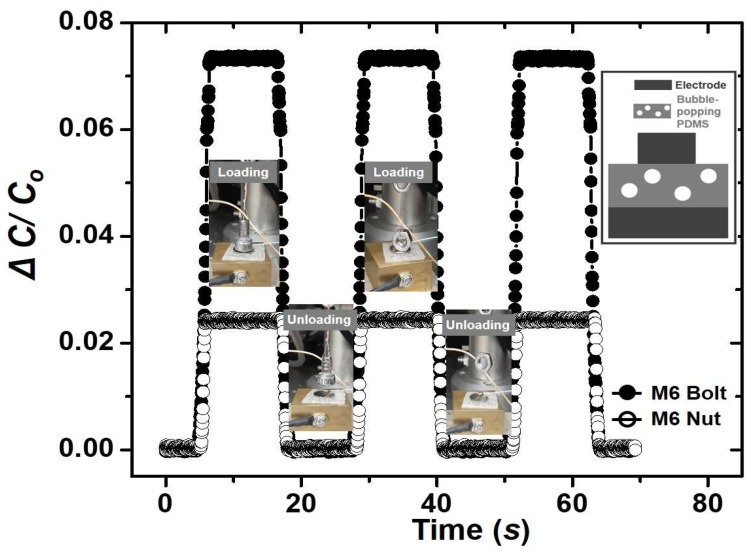
Capacitance response plot for the dynamic loading and unloading cycles of a small M6 bolt and nut. Inset; the device structure.

A capacitive pressure sensor made from the bubble-popping PDMS in an optimal 10:1:0.5 composition was used to sense the dynamic loading and unloading pressure of a small M6 bolt (4.5 g) and a small M6 nut (2.5 g). As shown in Figure 4, a high signal-to-nose ratio was obtained in the pressure measurements, which shows the high sensitivity of the pressure sensor made of the bubble-popping PDMS.

## 4. Conclusions

In this study, we proposed a method to form pores inside the PDMS to increase sensitivity while maintaining linear response characteristics and to eliminate the effect of contact area changes that occur when force was applied. The sensitivity of the pressure sensor made of the bubble-popping PDMS was approximately 4.6 times better than that of the pressure sensor without pores. The pressure sensor made of the bubble-popping PDMS had a very high linear response to the external pressure changes. We also examined the ability of the pressure sensor made of the bubble-popping PDMS to detect the dynamic loading and unloading pressure of a small M6 bolt and nut. These results suggest that our pressure sensor, based on the bubble-popping PDMS and fabricated through a simple and inexpensive process using a PDMS and water emulsion, constitutes an effective device structure for enabling e-skins capable of detecting applied pressures or contact forces.

## Data Availability

The data that support the findings of this study are available from the corresponding author upon reasonable request.
